# Effects of Voice and Biographic Data on Face Encoding

**DOI:** 10.3390/brainsci13010148

**Published:** 2023-01-14

**Authors:** Thilda Karlsson, Heidi Schaefer, Jason J. S. Barton, Sherryse L. Corrow

**Affiliations:** 1 Human Vision and Eye Movement Laboratory, Departments of Medicine (Neurology), Ophthalmology and Visual Sciences, Psychology, University of British Columbia, Vancouver, BC V5Z 3N9, Canada; 2 Faculty of Medicine, Linköping University, 582 25 Linköping, Sweden; 3 Department of Psychology, Bethel University, St. Paul, MN 55112, USA

**Keywords:** semantic, memory, learning, person, facilitation

## Abstract

There are various perceptual and informational cues for recognizing people. How these interact in the recognition process is of interest. Our goal was to determine if the encoding of faces was enhanced by the concurrent presence of a voice, biographic data, or both. Using a between-subject design, four groups of 10 subjects learned the identities of 24 faces seen in video-clips. Half of the faces were seen only with their names, while the other half had additional information. For the first group this was the person’s voice, for the second, it was biographic data, and for the third, both voice and biographic data. In a fourth control group, the additional information was the voice of a generic narrator relating non-biographic information. In the retrieval phase, subjects performed a familiarity task and then a face-to-name identification task with dynamic faces alone. Our results consistently showed no benefit to face encoding with additional information, for either the familiarity or identification task. Tests for equivalency indicated that facilitative effects of a voice or biographic data on face encoding were not likely to exceed 3% in accuracy. We conclude that face encoding is minimally influenced by cross-modal information from voices or biographic data.

## 1. Introduction

There are many routes to recognizing people. Faces, voices, names, and information such as biographic data or episodic memories about someone can all serve as cues to prompt a sense that a person is familiar or lead to specific identification [1,2,3,4]. While these very different cues likely have distinct representations in the neural system, interactions between these forms of information are also likely, to generate the integrated experience of recognizing a person. How and where such integration occurs remains a topic of debate, not only for person recognition, but also for our experience of the world in general [5,6,7].

There is empiric behavioral evidence for cross-modal interactions between different forms of person information, though this is admittedly mixed. (Note: strictly speaking, the term cross-modal should refer to interactions between two stimuli perceived through different sensory modalities, such as sound and sight. Names and biographic data are verbally mediated conceptual data that can be transmitted through spoken or written material. Nevertheless, ‘cross-modal’ has been used less stringently to refer to interactions that involve such information, and we will continue that usage) A familiar face with its spoken name is recognized more accurately and rapidly than either the face or name alone, and functional imaging with multi-voxel pattern analysis suggests that this face-name integration is accompanied by effects in the posterior superior temporal sulcus and middle temporal gyrus (Li, Wang [8]). Recognition of a familiar voice is faster and more accurate when it is combined with its moving face than when heard in isolation [9]. On the other hand, another study found that the recognition of familiar people by a combination of their face and voic was no more accurate than for the face alone, though more accurate for either than for the voice alone, and none of the three conditions differed in response time [10].

There are also congruency effects, in that two pieces of information about the same person lead to better performance when they are correctly paired (i.e., from the same person) than when they are not (i.e., from different people). This form of interaction has been shown between familiar faces and spoken names (Li, Wang [8]), and between familiar faces and voices [9]. Similar congruency effects can occur for recently learned people, but this has been found only for the combination of faces and voices, not between names and either faces or voices [11,12]. It was speculated that this may reflect the fact that faces and voices are forms of perceptual representations and their identification processes share a right hemispheric predominance, possibly converging in the right anterior temporal lobe, while names and other verbal semantic information about people are more conceptual representations, with a left hemispheric predominance [13].

These studies generally establish the presence of cross-modal interactions during a retrieval operation, when a subject is asked to recall or recognize the person or stimulus. What this implies about interactions during encoding (the learning phase) is not clear. Encoding is necessary for later retrieval, and one might assume that face–voice interactions during retrieval mirror face–voice interactions during encoding, but this is not necessarily true. For example, one can envisage instances of temporally dissociated encoding: a subject could learn the voice of a famous person on the radio and their face in photographs, yet this may still plausibly lead to face-voice interactions during retrieval. Even when two stimuli are learned concurrently, later retrieval interactions may not be evidence that encoding of one had been modulated by the presence of the other: concurrent learning may have created contextual links rather than modifying representations. Although they likely operate on the same representations, encoding and retrieval processes differ [14] and one cannot assume that effects on one also apply to the other.

In the current report, we focused on the effects of cross-modal interactions during the encoding phase, to see if this enhances the later (unimodal) recognition of those faces. Prior evidence relevant to this issue is mixed. Some prior studies of encoding interactions involving face identity have suggested that face encoding is enhanced when the name or other semantic information is present [15,16,17]. However, other studies examining the facilitative effect of cross-modal interactions have not found any improvement of face encoding by hearing the person’s voice [18,19]. In the reverse direction, looking at whether faces enhance the encoding of other information, one study found that voice identification was more accurate when the voice had been learned with a face [20], but others did not [21,22]. A few even showed a disadvantage when the face was present while the voice was being learned [18,19]. This was termed ‘*face overshadowing*’ and attributed to the effects of divided attention.

Our goal was to examine whether other identifying non-face stimuli modulated the encoding of faces. We asked whether face learning was more effective when faces were accompanied by the additional cues of the voice or biographic data, rather than if faces were learned in isolation, and whether the combination of both voice and biographic cues was an even more effective aid to the encoding of faces. Given that congruency effects in recall have been reported at retrieval between faces and voices but not between faces and names [11,12], we hypothesized that voices may have a greater influence on face processing than biographic data, additional verbal semantic information besides the name. However, it is also possible that biographic information may have a greater influence on face processing [15,16,17] than voice information does [18,19]. We studied effects on both face familiarity, which likely reflects the formation of a face representation in visual memory, as well as face identification by name, indicating the formation of specific face–name associations at a later multimodal stage of encoding.

To advance on prior work and control for the possibility that any additional verbal information present during face encoding either facilitates or overshadows facial attention, we included a control condition in which faces were encoded with a cross-modal generic voice that provided non-biographic verbal information. To minimize any overshadowing, which may be a transient early effect [23], we used several cycles of relatively long durations of learning. We also used dynamic rather than static faces in the interests of ecological validity. This also allowed us to synchronize character’s voices with their faces, allowing for a possible contribution of audiovisual sensory integration [9], rather than just an interaction between stimuli that are concurrent but not necessarily synchronized.

## 2. Materials and Methods

### 2.1. Subjects

Forty-three subjects were recruited in total. Two were excluded because of poor accuracy for face familiarity (59%, 53%, chance being 50%) and a third because of low accuracy for face identification (23%, chance being 25%). The final forty subjects who participated (31 females and 9 males) ranged in age from 19 to 40 years (mean age = 27.4, s.d = 6.34). Subjects were assigned to one of four groups of ten subjects in a counterbalanced manner, so that the groups did not differ in age (F_(3, 36)_ = 2.24, *p* = 0.1). Power analyses indicated that to find a facilitative effect of 10%—chosen since a previous study (16) found a 13% accuracy increase in familiarity and a 9.1% increase in identification—with power of 0.80 at an alpha level of 0.05, we would need a sample size of nine per group.

All individuals were fluent in English and had at least ten years of exposure to Caucasian faces. They had normal or corrected-to-normal visual acuity, no history of neurological disorders and no self-reported problems with face recognition, memory, or hearing. Written informed consent was obtained from all the subjects and $10 CAD per hour of participation was given as compensation. The Institutional Review Boards of the University of British Columbia and Vancouver Hospital approved the protocol, and all subjects gave informed consent in accordance with the principles of the Declaration of Helsinki.

### 2.2. Stimuli

*Audiovisual Clips*. The stimulus set consisted of 48 faces, half male and half female, of actors ranging in age between 20 and 30 years of age. We created audiovisual clips of these actors with a Canon Rebel Ti3 Camera in a room with consistent lighting. To eliminate extraneous cues to identity, actors removed glasses or jewelry and were excluded if they had facial tattoos or piercings. All actors were Caucasian and fluent in English. Female actors were instructed not to wear heavy makeup and male actors removed facial hair prior to filming. All the actors wore identical black T-shirts and covered their hair with a black cap. The same black background was used for filming all the actors [11].

Three separate clips were filmed for each actor. In the *biographic* clips, actors stated their character’s name, age, where they were from, occupation, hobbies and other interesting facts. (e.g., “Hi, my name is Aiden and I’m 24 years old. I grew up in Vancouver but spent a year of university studying abroad in Barcelona. I’m doing a Master’s in Math right now and tutoring on the side. I also play soccer for fun at the University.”) Similar but unique types of information were given for each character. In the *script-reading* clips, actors read several paragraphs from a book about a hiking expedition. All actors read the same paragraphs. In the *looking around* clips, the actor silently looked around the room in a casual manner. The actors readjusted their shirt and cap between the filming of each clip, to prevent inadvertent pictorial cues to identity.

Audiovisual clips were edited to the correct length using Adobe Photoshop CC (2015.0) and rendered at 30 frames per second. Faces in the learning and testing phases spanned on average 12.5° (s.d. 0.6) of visual angle in width between external cheek contours and 10.1° (s.d. 0.7) in height, from the top of the head to the chin. To remove background noise and isolate the audio, Audacity 2.1.1 was used to first select a segment of the background noise as the noise profile and then apply a noise reduction of 12 dB at sensitivity of 6.00 to all audio clips. To minimize inadvertent cues from minor variations in zoom or background lighting, we edited the clips in one of several ways. Using a random number generator to determine which one alteration to apply to a clip, the following edits were made: crop from above, crop from below, crop from the right, crop from the left, zoom in, brighten midtones, darken midtones, darken all levels, decrease brightness 30%, increase brightness 30%, increase brightness 60%, or no edit.

*Biographic* and *script-reading* clips were trimmed to 20 s each and the *looking around* segment was divided into two different 15 s clips. The *biographic* and *script-reading* clips were edited so that the actor’s name appeared under the face in the neck region for the duration of the clip. The name appeared in Times New Roman font, size 24 in white with a black outline. The *biographic* and *script-reading* audiovisual clips were used in the learning phases of different experiments. The *looking-around* video-clips did not show the name and were used in the testing phases of all experiments. A pilot study was performed to ensure that the accuracy of face learning did not differ between the visual portions alone of the *biographic* and *script-reading* audiovisual clips, and to determine the amount of training needed to produce performance with neither ceiling nor floor effects.

*Narrator voice-over audio-clips.* These were recorded using Blue Microphones, The Yeti (2014) on the cardioid setting. The audio files were trimmed to 20 s using Audacity 2.1.2. The voice-overs were recorded by either one female or one male narrator, both with a North American accent. These voice-over narrations related the same semantic information as either the *biographic* or *script-reading* clips. The gender of the narrator always differed from the character in the video clip (e.g., information about female characters was given by a male narrator) and the information was presented in the third person point-of-view. These two features were intended to make it clear that the voice was that of a narrator and not that of the character.

### 2.3. Design

**Figure 1:**
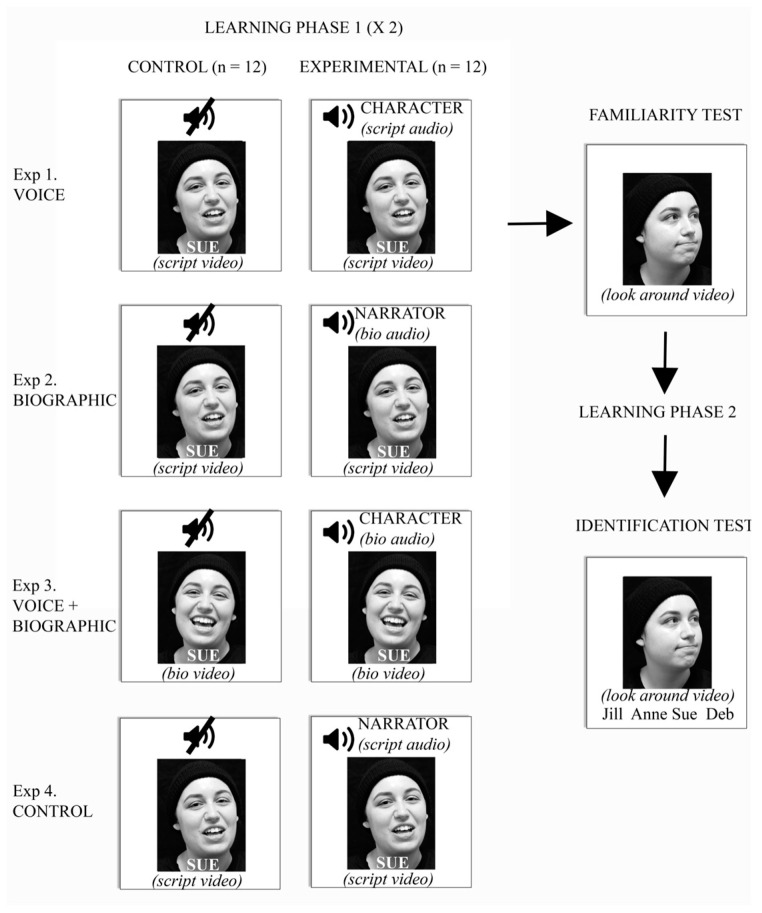
Methods. The learning phases for the four different experiments are shown on the left, with each row showing the control (face/name only) and experimental (multi-modal) conditions. Each box depicts the audio and visual components in each trial. If there was an audio-clip, either a narrator or the character could be talking, and relating either a generic script or specific biographic data (‘bio’) about the character. The video-portion of the clip (labeled below) could show the character’s face as they read the script or were giving their biographic data. After two cycles of learning, subjects then had a familiarity test, using different silent videos, of the character looking around. This was followed by a second learning phase of one cycle. The final phase was testing identification, by matching the silent ‘looking around’ face to the correct name in an array of four choices shown below the face.

A between-subjects design was used. Subjects were assigned in a counterbalanced order to one of four parallel experiments (Voice, Biographic, Voice + Biographic, and Control; see Figure 1). The four experiments differed from each other only in the learning phase. Each of the four experiments used the same set of twenty-four faces as targets, which were divided into sets A and B. For half of the subjects in each experiment, set A was learned without the sound (i.e., video-clip showing face and name only). This constituted the control ‘face/name’ learning condition. Set B included the auditory information appropriate to that experiment, and these were the multi-modal learning conditions. This assignment of sets A and B was reversed for the second half of the subjects, with subjects randomly assigned to the groups. The remaining 24 characters that were not used during the learning phase were used as distractors in the familiarity test.

In the ***Voice*** experiment, *script-reading* audiovisual clips were used for both the control (face/name) and the experimental multi-modal conditions, but without sound in the control condition. Thus, in the multi-modal condition of this experiment, face-learning was accompanied by the voice of the character, but the voice did not impart any biographic information.

In the ***Biographic*** experiment, subjects saw the visual portion of the *script-reading* clips. The control (face/name) condition again had no sound, but the experimental multi-modal condition also presented the auditory voice-over of the generic narrator giving the introductory biographic information about the character. Thus, in the multi-modal condition, face-learning was accompanied by biographic data about the character, but not by their own voice.

In the ***Voice + Biographic*** experiment, we used *biographic* audiovisual clips, with the sound removed in the control (face/name) condition but included in the experimental multi-modal condition. Thus, in the multi-modal condition, face-learning was accompanied by both the voice of the character *and* their biographic data.

In the ***Control*** experiment, we presented the visual portion from the *script-reading clips,* as in the ***Voice*** and ***Biographic*** experiments. The control (face/name) condition again had no accompanying auditory information, but the experimental multi-modal condition had a generic narrator of the opposite gender reading the *script* about the hiking expedition. This condition served to examine if the presence of a generic voice with non-person-related semantic content during face encoding had any effect, in particular, a detrimental one that could reflect an effect of divided attention.

### 2.4. Procedure

Experiment Builder 1.10.1630 was used to program the experiment (www.se-research.com, accessed on 3 June 2016), which was viewed on a ViewSonic screen and with the auditory component transmitted through two Dell A00 speakers placed on each side of the screen.

Each subject completed four phases of the experiment: Learning, familiarity, re-learning, and identification. The whole experiment took approximately 30 min.

*Learning phase 1*: Subjects were presented with 24 characters, one at a time, and instructed to try to remember everything about the people presented, including not only their face and name, but also their voice and any biographic information if presented. All audiovisual clips in the learning phase were 20 s long. Subjects could not truncate the clips in the learning phase: only after a clip ended were they allowed to press the space bar to start the next clip when they were ready. The learning phase had two blocks. Both presented the same clip of each of the 24 characters once, but in a different random order. Subjects could take a short break of a minute or less between the two blocks. Thus, subjects had a total of 40 s of learning for each character in this first phase.

*Familiarity test:* This presented the 24 target faces that had been learned and the 24 distractor faces in a random order, without sound. Two different *looking-around* video-clips were shown for each face. These test clips did not show the character’s name. One video-clip for each target and distractor face was shown in random order in a first block, followed by a second block that showed the second video clip for each of the 48 faces, in a different random order, for a total of 96 trials. Subjects were not aware of the separate blocks but rather were told that they could take a break of a minute or less between the blocks. The subject’s task was to press “f” on the keyboard if the face was familiar (i.e., had been seen in the learning phase) or “u” if it was unfamiliar. Each trial started with a blank screen with a fixation cross for 200 ms followed by the *looking-around* video-clip of the face, which lasted 15 s. They were asked to respond as rapidly and accurately as possible. If a response took longer than 4 s, a screen appeared prompting the subject to choose faster. No feedback was given.

*Learning phase 2:* After completing the familiarity task, the subjects were given a second learning phase. The material was identical to those in the first learning phase, but now with only one block that showed each of the 24 target characters once in a random order, instead of two blocks. Combining the two learning phases, subjects had a total duration of 60 s of learning per character before the identification test.

*Identification test*: This presented the same two *looking-around* video-clips of the 24 target stimuli that were seen in the familiarity test, but not the 24 distractor faces. These clips were again divided into two blocks and shown in random order for a total of 48 trials. Subjects could take a pause of a minute or less between the two blocks. Below each video-clip of a face we showed four gender-appropriate names. Thus, for video-clips of female faces, four female names were presented; the target character’s name and the names of three of the remaining 11 female characters who had been learned, chosen randomly. On each trial, the location of the correct name was randomized between the four possible locations. Subjects were instructed to match the name to the face of the character by clicking on it with the mouse. There was no time limit, and no feedback was given.

### 2.5. Analysis

For each subject, we calculated their accuracy for familiarity and identification judgments. For the familiarity test we also calculated their mean response time for correct trials, excluding those trials whose response times were more than 3 standard deviations from the mean. We did not analyze response times for identification because these were prolonged, generally around 4 s or more, and confounded by the time needed to move the mouse between the different choices on the screen.

On each of the three outcome variables, we conducted a 2 × 4 mixed factor ANOVA with condition (control face/name, multi-modal) as a within-subjects factor and experiment (voice, biographic, voice-biographic, and control) as a between-subjects factor, separately for familiarity and identification results. We also performed planned *a priori* pair-wise comparisons between multi-modal and control conditions of each experiment, using 2-tailed paired-sample student’s t-tests with Bonferroni correction for multiple comparisons (critical *p* = 0.0125).

## 3. Results

For all tests and all conditions combined, accuracy was 0.73 (s.d. 0.12) for the familiarity test, where chance performance was 0.50, and 0.76 (s.d. 0.14) for the identification test, where chance was 0.25. Thus performance was neither at ceiling nor at floor. The overall average of the mean response times for familiarity judgments was 1226 ms (s.d. 264).

**Figure 2:**
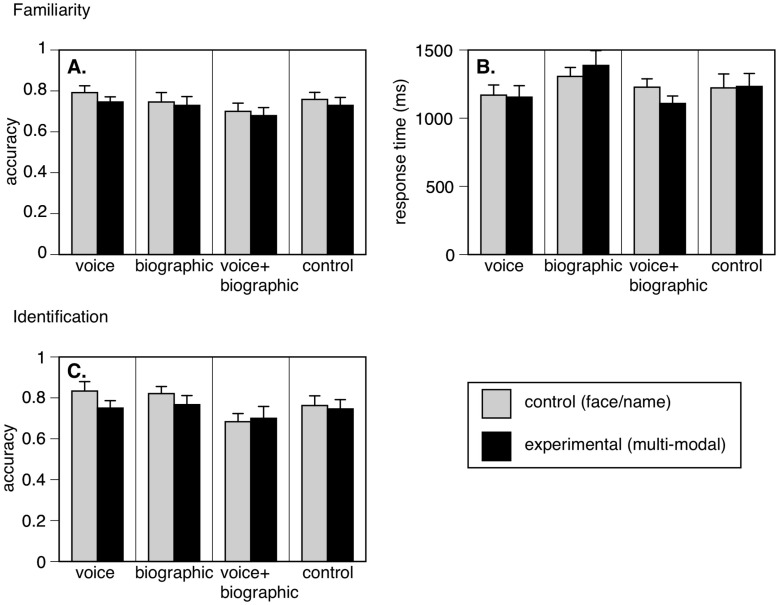
Results (**A**) Familiarity accuracy, (**B**) Familiarity response time, and (**C**) Identification accuracy. Each graph shows results for the four experiments separately (Voice, Biographic, Voice + Biographic, and Control). The bars compare mean performance of learning the face alone with learning the face accompanied concurrently with the additional information appropriate to that experiment. Error bars show one standard error.

***Familiarity Task***: For accuracy (Figure 2A), there was no main effect of the experiment (F_(3, 36)_ = 1.08, *p* = 0.37, η^2^ = 0.08). Thus, all four subject groups learned the stimuli to a similar degree. There was no main effect of condition (F_(3, 36)_ = 1.83, *p* = 0.18, η^2^ = 0.05) nor any interaction between condition and experiment (F_(3, 36)_ = 0.1, *p* = 0.96, η^2^ = 0.008). Examining the planned *a priori* pair-wise comparisons, if anything accuracy was slightly better for the control (face/name) conditions; and no linear contrast was significant (all *p*-values > 0.29).

For reaction time (Figure 2B), the ANOVA again showed no main effect of the experiment (F_(3, 36)_ = 1.22, *p* = 0.32, η^2^ = 0.09). There was no main effect of condition (F_(1, 36)_ = 0.14, *p* = 0.71, η^2^ = 0.004) nor an interaction between condition and experiment (F_(3, 36)_ = 2.04, *p* = 0.13, η^2^ = 0.145). The paired-sample t-tests did not show a difference between control (face/name) and experimental multimodal conditions in any of the four experiments (all *p*-values > 0.053).

***Identification Task***: For accuracy (Figure 2C), there was no main effect of experiment (F_(3, 36)_ = 1.42, *p* = 0.25, η^2^ = 0.106). There was a trend to an effect of condition (F_(1, 36)_ = 3.24, *p* = 0.08, η^2^ = 0.083), but this was due to slightly better rather than worse accuracy on control face/name than on multi-modal trials. There was no interaction between condition and experiment (F_(3, 36)_ = 1.23, *p* = 0.31, η^2^ = 0.093). Two-tailed paired-sample t-tests did not show a difference between control (face/name) and experimental multimodal conditions in any experiment (all *p*-values > 0.07).

Given that we cannot reject the null hypothesis that a second cue had no effect on face recognition, we performed tests for equivalence between face/name and multi-modal conditions [24]. Considering all three experimental groups together (n = 30), the probability was less than 0.05 that facilitative effects would be greater than 0.025 in accuracy or 92 ms in response time for familiarity, and 0.022 in accuracy for identification.

## 4. Discussion

We found no evidence that voice, biographic data, or the combination of the two presented concurrently with faces at encoding facilitated the later recognition of newly learned faces, in either familiarity judgments or identification through name–face matching. Although the number of subjects in each experiment was modest, the results were consistent across all four experiments, in a total of 40 subjects. The results are also consistent with those of a companion study on face or voice facilitation of biographic data encoding, using similar methods in another 31 subjects [25]. Our test for equivalency suggested that any facilitative effects would be fairly minimal, in the range of a 2 to 3% gain in accuracy. In addition, our fourth control experiment showed that, for the number of cycles and duration of learning used in our study, overshadowing effects from voices or biographic data were negligible.

While a number of studies have examined the impact of faces on voice learning, with mixed results [18,19,20,21,22,23], only two have looked at the reverse, whether a concurrent voice improves the learning of static faces in photographs. One showed that 60 s of face learning was not improved by voices, when tested later in recognition of the face in a line-up [18]. A second study found no difference in an old/new face-familiarity task after 8 s of learning [19]. Our study had 40 s of learning before familiarity testing and 60 s before identification testing, and similarly showed no effect, even despite using dynamic faces that allowed for audiovisual integration.

How about semantic information: does this aid face encoding compared to learning faces alone? Semantic information about people includes both names and biographic data, but these may not be equivalent. While some treat names as a subset of biographic data [26], other cognitive models consider name retrieval to be a stage that follows biographic data processing [27], or envision separate name and biographic processing modules [28]. This may be consistent with evidence that name retrieval can be impaired independently of the ability to access biographic data [29]. Hence, it may be important to consider the effects of biographic data and names separately.

One study had 15 subjects learn 20 static faces and found that the accuracy of a familiarity judgment improved by 0.04 to 0.06 if the face was accompanied by a written name [15]. In their fourth experiment, they obtained modest gains of 0.03 when the written information gave an occupation rather than a name. Both our control and experimental conditions presented the face and the name, with the aim of later testing the more stringent task of identification through face–name matching. Hence we controlled for any effect from the name. Rather, the experimental manipulations in our second and third experiments were the presentation of several items of biographic data including occupation, in auditory rather than written form. Given that our test of equivalency suggests that any facilitative effect would be less than 0.025, our results are comparable with their finding of a small effect of 0.03 from occupational data.

How about the combination of voices and semantic information, as in our third experiment? One prior study had 12 subjects learn static faces, 20 alone and 20 paired with 20 voices that gave the name and one biographic detail. With very brief learning durations of three cycles of 300 ms exposure per face, face familiarity was 13% better with the additional information [16]. This study was later criticized for using the same pictures in the learning and test phases, because responses could reflect image rather than person recognition [17]. A second study had 24 subjects learn dynamic faces over 30 s, 36 alone and 36 paired with 36 non-synchronized voices that related the character’s name and several biographic details [17]. Having the additional information resulted in better d’, though the effects on accuracy were mixed. Both of these studies included name, biographic data, and the voice in their experimental manipulation. Given the strong effect of name alone found by [15], the results of these two reports cannot establish whether there are additional enhancing effects due to voice or biographic data. Our experiments isolated the contributions of the voice and/or biographic data since the name was presented in both the control and experimental conditions, and we found negligible effects.

Overall then, with one exception [16] most of the studies above suggest either no or very modest effects of voices or biographic data on face encoding, consistent with our results. If this is true, how are congruency [11,12], cueing [30] and other facilitative effects at retrieval explained? One possibility is suggested by two studies [21,22]. These found that enhanced voice familiarity by concurrent learning with faces was only evident when the test phase presented, not the voice alone, but the voice and the face together. Since the same faces appeared with either the target or the distractor voices, they were not informative as to which was the correct answer. Rather, they were a contextual cue that prompted recognition of the correct face. If so, having a concurrent second cue at learning may not enhance encoding of the first cue, but may establish an associative connectivity between the two representations that can facilitate later retrieval.

Other explanations for the lack of facilitative effects in our study need to be considered. One possibility is that facilitative effects may vary with the duration of learning. One study of voice learning found detrimental effects from a concurrent face with 6 to 12 s of learning, which was attributed to divided attention from ‘face overshadowing’, but facilitative effects with 29 to 35 s of learning [23]. Though a similar temporal dynamic was not found in another study [21], to guard against overshadowing we had subjects learn faces for 40 s before the familiarity test and for 60 s before the identification test. While it is not known whether voice overshadowing of faces occurs, we included a fourth control experiment to determine if this could be a factor in our results. This did not show any degradation of face learning when a narrator’s voice related verbal information without biographic data. Lack of overshadowing from voices on faces may have several explanations, besides the possible effects of the temporal dynamics discussed above. Overshadowing is more likely from dominant stimuli such as faces when weaker ones such as voices are being learned, rather than vice versa [18,19]. This may reflect the ’inverse effectiveness principle’, that strong cues are more likely to enhance performance with weak stimuli than vice versa [31]. Additionally, if overshadowing reflects competition for attention, it may be that a generic narrator’s voice relating irrelevant information may not demand much attention, and thus not cause voice overshadowing in our fourth control experiment. Nevertheless, lack of voice overshadowing is supported by the fact that we also did not find a decrement in performance when the character’s voice or biographic data were presented, to which subjects were instructed to attend.

The ’inverse effectiveness principle’ [31] also suggests that facilitative effects of other person-related stimuli on face encoding could be minimal simply because humans are much more proficient at face recognition. Numerous studies show that face recognition is superior to voice recognition [19,21,32,33]. From this one could predict that faces might facilitate voice encoding, but that face encoding would not be facilitated by voices. To date, there has not been a study that has used similar methods to directly compare the two.

Nevertheless, human proficiency with faces does not exclude the possibility of any facilitative effects. Names with or without other semantic or voice data appear to enhance face familiarity [15,16,17]. In our study, names were presented in both control and experimental conditions so that their effects were controlled. One might question whether including names optimized face encoding to a point where further improvement was unlikely, which could be addressed by a study of face familiarity in which names were omitted. However, we note that mean familiarity accuracy in the control (face/name) conditions was less than 80%, and therefore not at ceiling.

Another possibility is that facilitative interactions in encoding only occur as a specific effect of certain stimuli. One study reported that the encoding of dynamic faces was better when these were presented with familiar than with unfamiliar names [34], while another showed that the familiarity judgments were better for faces paired with distinctive rather than with typical voices [35]. Since neither of these studies included a condition in which the face was learned alone, it is not clear whether the differences represent a facilitative effect of the more effective stimulus or a detrimental effect of the less effective one.

There are other observations that are relevant to our findings. A study of the ‘depth’ of encoding of faces found that having subjects engage in judgments of character or what hobby a person was likely to have while they memorized faces did not improve face familiarity above a condition of natural self-directed attention [36]. Thus, neither the internally generated semantic connections in that study nor the externally provided semantic data in our study facilitated familiarity. Conversely, we have recently shown with a similar experimental design that the encoding of biographic data shows little facilitation by a concurrent face and/or voice [25].

## 5. Conclusions

We find little evidence that face encoding is enhanced by either the person’s voice or biographic data, regardless of whether retrieval is tested by familiarity or identification through face-name matching. This suggests that the encoding stage of face learning is relatively impervious to modulation by other information from other modalities. Hence, the multi-modal benefits seen in the retrieval phase of other studies may be due not to multimodal enhancement of encoding, but rather to other effects, such as the formation of connectivity patterns through associative learning, which can boost retrieval through contextual cues.

## Data Availability

Data are available in a publicly accessible repository that does not issue DOIs. They can be found in TKarlsondata.xlsx at https://osf.io/ptu6q/files/osfstorage, accessed on 26 October 2022.
